# Enhanced angiogenic potential of adipose-derived stem cell sheets by integration with cell spheroids of the same source

**DOI:** 10.1186/s13287-022-02948-3

**Published:** 2022-06-28

**Authors:** Jiashing Yu, Yi-Chiung Hsu, Jen-Kuang Lee, Nai-Chen Cheng

**Affiliations:** 1 Department of Chemical Engineering, College of Engineering, National Taiwan University, 1 Sec. 4, Roosevelt Rd., Taipei 106, Taiwan; 2 Department of Biomedical Sciences and Engineering, National Central University, 300 Zhongda Rd., Taoyuan 320, Taiwan; 3 Department of Medicine, National Taiwan University Hospital and College of Medicine, 7 Chung-Shan S. Rd., Taipei 100, Taiwan; 4 Department of Surgery, National Taiwan University Hospital and College of Medicine, 7 Chung-Shan S. Rd., Taipei 100, Taiwan

**Keywords:** Adipose-derived stem cell, 3D culture, Cell spheroid, Cell sheet, Angiogenesis

## Abstract

**Background:**

Adipose-derived stem cell (ASC) has been considered as a desirable source for cell therapy. In contrast to combining scaffold materials with cells, ASCs can be fabricated into scaffold-free three-dimensional (3D) constructs to promote regeneration at tissue level. However, previous reports have found decreased expression of vascular endothelial growth factor (VEGF) in ASC sheets. In this study, we aimed to integrate ASC spheroids into ASC sheets to enhance the angiogenic capability of cell sheets.

**Methods:**

ASCs were seeded in agarose microwells to generate uniform cell spheroids with adjustable size, while extracellular matrix deposition could be stimulated by ascorbic acid 2-phosphate to form ASC sheets. RNA sequencing was performed to identify the transcriptomic profiles of ASC spheroids and sheets relative to monolayer ASCs. By transferring ASC spheroids onto ASC sheets, the spheroid sheet composites could be successfully fabricated after a short-term co-culture, and their angiogenic potential was evaluated in vitro and in ovo.

**Results:**

RNA sequencing analysis revealed that upregulation of angiogenesis-related genes was found only in ASC spheroids. The stimulating effect of spheroid formation on ASCs toward endothelial lineage was demonstrated by enhanced CD31 expression, which maintained after ASC spheroids were seeded on cell sheets. Relative to ASC sheets, enhanced expression of VEGF and hepatocyte growth factor was also noted in ASC spheroid sheets, and conditioned medium of ASC spheroid sheets significantly enhanced tube formation of endothelial cells in vitro. Moreover, chick embryo chorioallantoic membrane assay showed a significantly higher capillary density with more branch points after applying ASC spheroid sheets, and immunohistochemistry also revealed a significantly higher ratio of CD31-positive area.

**Conclusion:**

In the spheroid sheet construct, ASC spheroids can augment the pro-angiogenesis capability of ASC sheets without the use of exogenous biomaterial or genetic manipulation. The strategy of this composite system holds promise as an advance in 3D culture technique of ASCs for future application in angiogenesis and regeneration therapies.

**Supplementary Information:**

The online version contains supplementary material available at 10.1186/s13287-022-02948-3.

## Introduction

The therapeutic potential of stem cells is enormous for applications in tissue engineering and regenerative medicine [1]. Mesenchymal stem cell (MSC) represents an important category of stem cells with the capability of self-renewal and multi-lineage differentiation [2]. Among MSCs from different tissue origins, adipose-derived stem cells (ASCs) can be harvested abundantly and safely by minimally invasive surgical procedures, such as liposuction surgery [3, 4]. ASCs can express the pluripotency markers, including Sox2, Oct4, and Nanog, to increase their self-renewal potential and suppress the spontaneous differentiation of these cells [5]. Moreover, ASCs also secrete various growth factors or cytokines that can promote angiogenesis, such as vascular endothelial growth factor (VEGF), hepatocyte growth factor (HGF), and fibroblast growth factor-2 (FGF2) [2]. Hence, ASCs are considered good candidates for a broad range of cell-based therapeutics targeting ischemic diseases, such as myocardial infarction and peripheral arterial disease [6].

Comparing to the conventional monolayer culture, the three-dimensional (3D) culture system resembles the natural MSC microenvironment in vivo and provides enhanced cell–cell and cell–extracellular matrix (ECM) interactions, leading to improved biological functions of MSCs [7]. For example, cell sheet fabrication as a 3D culture technology has shown potential in their tissue regenerative effects [8, 9]. By supplementing ascorbic acid 2-phosphate (A2-P) in the culture medium, we were able to fabricate ASC sheets with abundant deposition of ECM in conventional polystyrene culture plates. Particularly, enhanced stemness and transdifferentiation capabilities were found in ASCs upon sheet formation [9]. Enhanced wound healing and tissue regeneration were also noted after applying ASC sheets in an animal wound model. The mechanism was attributed to the enhanced secretion of antifibrotic and immunomodulatory cytokines from ASC sheets [10].

Despite the multiple advantages of cell sheet, downregulation of *VEGF* was noted upon ASC sheet formation [11]. Several studies have suggested that VEGF is a key regulator of the paracrine effects of ASCs [12], so decreased VEGF secretion may hinder the regenerative effects of ASC sheets for ischemic tissues. To address this shortcoming, ASC sheets were attached to FGF2-tethered decellularized dermal matrix to enhance their angiogenic potential [13]. In another study, ASC sheets were transfected to express VEGF to restore perfusion and protect ischemic tissues [14]. However, the use of exogenous biomaterials, especially those modified with recombinant growth factors, may exhibit certain untoward side effects. The potential risk of tumorigenesis resulting from gene manipulation also renders the VEGF-transduced cell sheets difficult to be applied for clinical use. Hence, an approach with less biosafety concern is desired to resolve this issue.

Previous studies have revealed that another 3D culture format by spheroid formation is associated with upregulated genes and enhanced secretion of angiogenic growth factors in human ASCs [15, 16]. These findings are in line with several other reports that identify aggregation of MSCs from other tissues can enhance their therapeutic potentials, including angiogenesis [17–19]. Therefore, we hypothesized that seeding ASC spheroids onto ASC sheets is a straightforward approach to enhance the angiogenic potential of ASC sheets. In this study, we prepared size-controllable ASC spheroids using agarose microwell plates. Subsequently, ASC spheroids were seeded onto the ASC sheets, and they fused preliminarily upon further culture and were ready for cell transplantation shortly. In this composite 3D culture system, ASC spheroids reinforced the angiogenesis characteristics of ASC sheets, thereby promoting the overall potential of ASC sheets to treat ischemic tissues without the use of exogenous biomaterial or genetic manipulation.

## Materials and methods

### Isolation and culture of human ASCs

ASCs were isolated from the subcutaneous fat tissue of four nonsmoking, nondiabetic female donors with an average age of 45 y (32–57 y) and an average body mass index of 24.6 (21.0–26.6) as described previously [10]. The study protocol has been approved by the Research Ethics Committee of National Taiwan University Hospital, and the informed consent has been obtained from each donor of adipose tissue. Small pieces of subcutaneous fat tissue were finely minced and washed using phosphate-buffered saline (PBS; Omics Biotechnology, Taipei, Taiwan), followed by enzymatic digestion using collagenase type I (Gibco, Carlsbad, CA, USA) for 60 min. Pellets were suspended and plated with Dulbecco’s modified Eagle’s medium (DMEM)/F-12 (HyClone, Logan, UT, USA) supplemented with 10% fetal bovine serum (FBS; HyClone), 1% penicillin–streptomycin (Biological Industries, Kibbutz Beit Haemek, Israel) and 1 ng/mL FGF2 (R&D systems, Minneapolis, MN, USA). The cells were cultured in a humidified atmosphere with 5% CO_2_ at 37 °C, and the medium was changed every 2–3 day. Upon reaching 90% confluence, the cells were detached using 0.05% trypsin–EDTA (Biological Industries) and re-plated until the third or fourth passage for further experiments. Human ASCs from each donor were pooled to become a single population based on the comparable surface marker expression and differentiation potential demonstrated in each ASC clone. These cells, which were constantly cultured on tissue culture plates, were referred as monolayer ASCs.

### Preparation of agarose microwell plates and ASC spheroids

The ASC spheroids were generated as previously described with certain modification [20]. As a non-cell-adhesive substrate, sterile solution of 2% agarose (UniRegion Biotech, Taipei, Taiwan) in PBS was autoclaved and dispensed onto sterile MicroTissues 3D Petri dishes (Merck, Darmstadt, Germany) following the manufacturer’s instruction. After the gelation of agarose molds at room temperature (about 5–7 min), they were gently detached from the petri dishes and immersed in sterile PBS. To equilibrate the petri dishes, they were immersed in a basal medium consisting of DMEM-high glucose (DMEM-HG; HyClone), 10% FBS and 1% penicillin–streptomycin for 15 min before use. Each agarose mold exhibited a structured surface with an array of 256 inverted circular recesses (depth 400 μm, diameter 800 μm). ASCs were seeded onto equilibrated agarose molds at different densities to achieve an average of 2000, 4000 or 8000 cells per microwell. The cells were incubated in the basal medium for 7 days for spontaneous spheroid formation, while the medium was carefully refreshed every 2–3 days.

### Fabrication of ASC sheets and spheroid sheets

To create cell sheets, ASCs were seeded in tissue culture plates at a density of 2.5 × 10^4^ cells/cm^2^ and cultured for 7 days. The culture medium consisted of basal medium and 250 μM A2-P as previously described [9]. The culture medium was refreshed every 2–3 days. While ASC sheets were fabricated, ASC spheroids were also cultured in agarose molds. At day 7, all ASC spheroids from 256 microwells in one agarose mold were carefully transferred onto a prefabricated ASC sheet by inverted placement of the mold onto the ASC sheet. After 3 more days of culture, a composite spheroid sheet was formed, and it could be peeled off from the culture plates for further experiments.

### Microscopic images and histology

ASC spheroids consisted of different cell numbers were photographed by an inverted microscope (TS100; Nikon, Tokyo, Japan). The Ferret diameter of the cell spheroids was determined by tracing the periphery of each spheroid using ImageJ. For the electron microscopic study, ASC spheroids consisted of different cell numbers were seeded on µ-slides (ibidi, Fitchburg, WI, USA) overnight. After washing ASC spheroids and ASC sheets thoroughly and fixed in 4% paraformaldehyde for 20 min, they were dehydrated by gradual change of concentrated ethanol, followed by lyophilization. The specimens were subsequently sputter coated with platinum and examined using a scanning electron microscope (JSM-6700F; JOEL, Tokyo, Japan).

A second harmonic generation microscopic system was employed to examine ECM deposition within ASC sheets as previously described [21]. A wavelength-tunable Ti/sapphire laser pumped by a diode-pumped solid-state laser (Spectra Physics, Mountain View, CA, USA) was used as the excitation source. The 880 nm output laser system was guided toward a modified upright microscope (BX51WI; Olympus, Tokyo, Japan). The excitation source was beam expanded and reflected toward the focusing objective by a primary dichroic beamsplitter (Semrock, Rochester, NY, USA). The laser power was set at approximately 70 mW on the tissue section to optimize image quality without laser ablation. The nonlinear autofluorescence signals were spectrally separated by bandpass filters of 434/17 nm and 510/84 nm (Semrock).

As for the histological analysis, ASC spheroids and spheroid sheets were fixed in 4% paraformaldehyde and then paraffin-embedded. Sections were cut perpendicular to the surface of the cell construct with a thickness of 5 µm. Paraffin-embedded sections were deparaffinized, rehydrated and stained with hematoxylin and eosin (H&E; Sigma, St. Louis, MO, USA).

### RNA isolation and RNA sequencing analysis

Total RNA from monolayer ASCs, ASC spheroids and ASC sheets was isolated by TRIzol reagent (Invitrogen). RNA quantity and purity were assessed using a microvolume spectrophotometer (Biochrom, Cambridge, UK), and the quality was verified by agarose electrophoresis and a Bioanalyzer 2100 (Agilent Technologies, Santa Clara, CA, USA) with RNA 6000 LabChip kit (Agilent Technologies). RNA libraries were performed with reagents supplied in Agilent's SureSelect Strand-Specific RNA Library Preparation Kit and sequenced on Illumina NovaSeq6000 platform with 150 paired-end reads. Raw sequences were expected to generate 20M (million reads) per sample according to Illumina’s standard sequencing protocol.

The generated sequences went through a filtering process to obtain qualified reads. Low-quality reads containing adapters or ploy-N from the raw reads were trimmed or removed according to the quality score. The filtered reads were aligned to the reference genomes using Bowtie2 (version 2.3.4.1). Qualified reads after filtering low-quality data were analyzed using RSEM (RNA-Seq by expectation–maximization) for gene expression estimation. The gene expression level was calculated as TPM (transcript per million). For differential expression analysis, edgeR v3.5 was employed to perform statistical analyses of gene expression profiles. The reference genome and gene annotations were retrieved from Ensembl database. The raw sequencing data were submitted to the NCBI Sequence Read Archive with BioProject ID PRJNA742860.

### Functional enrichment analysis

After filtering out the low expressed genes which were less than 10 RPKM expression level in samples, differential expression genes were determined with 2× fold change compared with monolayer group (whether is upregulated or downregulated). The molecular relationships were generated using the core analysis showing significant (*p* < 0.05) association by Ingenuity Pathway Analysis software (IPA; QIAGEN, Redwood City, CA, USA). Canonical pathway analysis found by core analysis in IPA is given with a p value.

### Quantitative polymerase chain reaction (qPCR)

Total RNA was extracted using RNeasy Mini Kit (Qiagen, Valencia, CA, USA) according to the manufacturer’s protocol. After the RNA was isolated, complementary DNA (cDNA) was synthesized from the RNA using High-Capacity cDNA Reverse Transcription Kits (Applied Biosystems, Foster City, CA, USA). Real-time qPCR was performed with iQ SYBR Green Supermix (Bio-Rad, Hercules, CA, USA) using CFX Connect Real-Time PCR Detection System (Bio-Rad). The expression level was analyzed and normalized to glyceraldehyde 3-phosphate dehydrogenase (GAPDH) for each cDNA sample. The relative quantity (RQ) of gene expression was calculated by relative quantification based on threshold cycle number. The sequences of the gene-specific primers are shown in Additional file 1: Table S1.

### ASC spheroid labeling and immunofluorescence

ASCs labeled with PKH26 (Sigma) were subjected to spheroid formation process. After seeded on ASC sheet for 4 days, the spheroid sheet was fixed in 4% paraformaldehyde and treated with anti-CD31 (Abcam, Cambridge, MA, USA) overnight at 4 °C. After rinsing for three times, the secondary antibody was applied, followed by counterstaining with 4′,6-diamidino-2-phenylindole (DAPI; Sigma). Spheroid sheets were analyzed by a fluorescent microscope (Leica DMI 6000), and negative controls without primary antibodies were also prepared to rule out nonspecific labeling.

### The angiogenic potential of ASC-conditioned medium

Monolayer ASCs, ASC sheets and spheroid sheets were washed with PBS and supplied with serum-free DMEM-HG. The conditioned medium was collected after 48 h as conditioned medium. The concentration of HGF, VEGF and FGF2 in the conditioned medium was determined using relevant ELISA kits (R&D Systems). Data were expressed as the secreted factor per 10^4^ cells at the time of harvest.

Human umbilical vein endothelial cells (HUVECs) were seeded on μ-slides (Ibidi) coated with Matrigel (Corning, Corning, NY, USA) at a density of 8 × 10^3^ cells/well, and they were cultured in a mixture of endothelial basal medium (EBM; PromoCell, Heidelberg, Germany) and conditioned medium from experimental groups of monolayer ASC, ASC sheet and spheroid sheet with a volume ratio of 4:1. Tube formation assay was performed as previously described [11]. A mixture of EBM and DMEM-HG with a volume ratio of 4:1 served as a negative control, while endothelial growth medium 2 (EGM2, PromoCell) was used as a positive control. At 6 h, formation of tubelike structures was visualized by a phase-contrast microscope, and the images were analyzed using ImageJ.

### In ovo angiogenesis assay

We employed the chick embryo chorioallantoic membrane (CAM) model as an in ovo assay for angiogenesis evaluation. Briefly, fertilized chicken eggs were incubated at 37 °C with 60% humidity. On day 3, a circular window was made on the upper side of the egg to evaluate the embryo viability. On day 7, ASC sheets or spheroid sheets were placed onto the CAM through the open window. The opening window in the shell was sealed with Tegaderm (3M, St. Paul, MN, USA) to prevent dehydration and contamination, and the eggs were placed into the incubator at 37 °C for 3 more days. On day 10, the embryos were infused with 4% paraformaldehyde and placed at − 80 °C overnight. ASC sheets or spheroid sheets with adjacent CAM tissues were removed and transferred to 6-well plates containing 4% paraformaldehyde. Untreated CAM tissues were used as controls. The CAM specimens were photographed, and the blood vessels were quantified by measuring the capillary area and counting the capillary branch points using ImageJ. Moreover, the immunohistochemical staining of the CAM sections was performed using anti-CD31 antibody as described previously [22]. The CAM sections were photographed under a microscope, and the ratio of CD31-positive area was quantified using ImageJ.

### Statistical analysis

All measurements are presented as means ± standard deviation. Statistical significance was evaluated using one-way ANOVA followed by the Tukey’s post hoc test. When Tukey’s test was used, each group was compared to all other groups in the experiment. All statistical analyses were performed using GraphPad Prism 7 (La Jolla, CA, USA), and statistically significant values were defined as *p* < 0.05.

## Results

### Fabrication of ASC spheroids and sheets

**Fig. 1:**
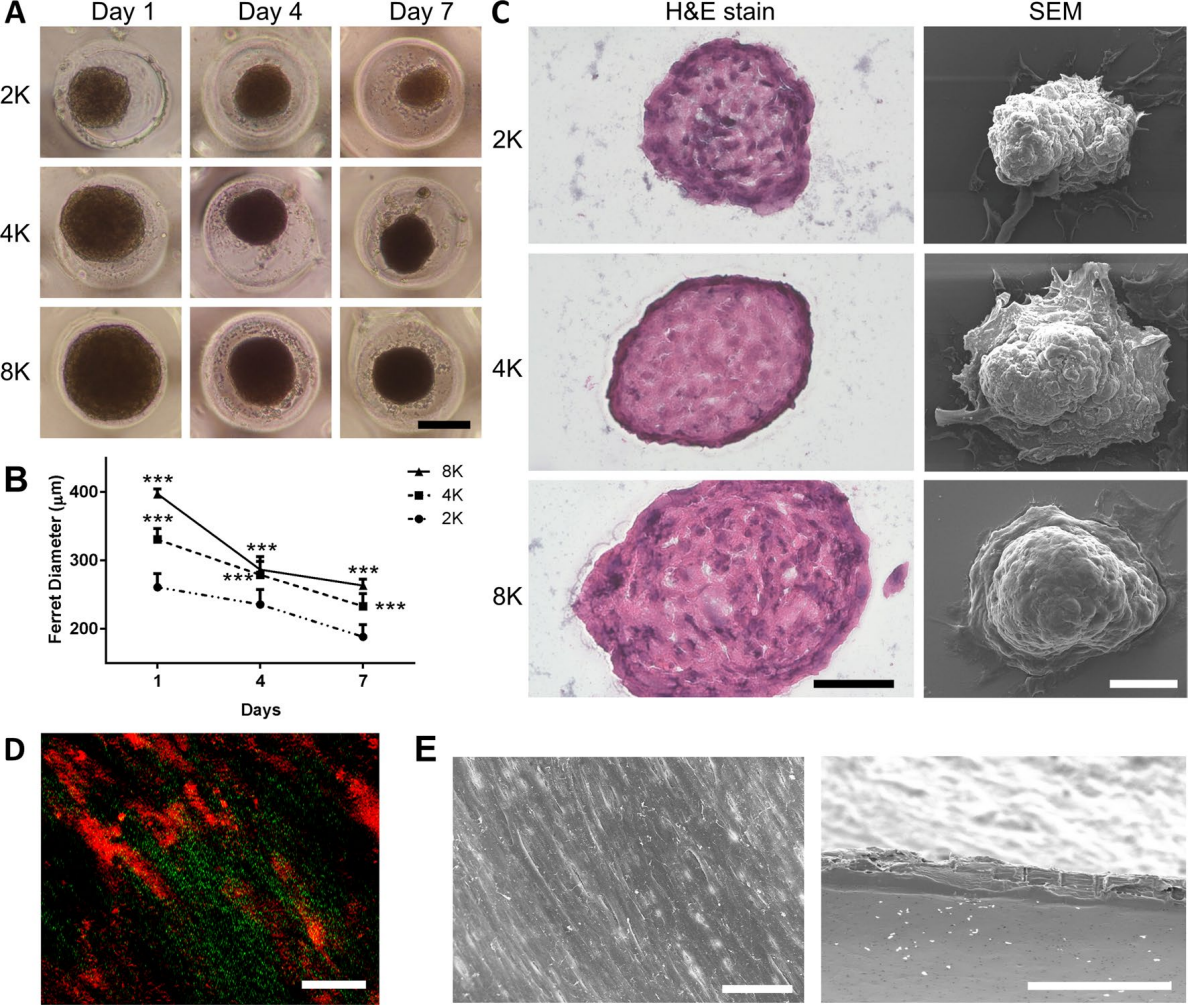
ASCs cultured in microwells at different seeding densities formed spheroids of different sizes in 7 days. **a** Representative microscopic images of ASC spheroids formed by respective cell number (2K, 4K, and 8K; K: 1 × 10^3^ cells; scale bar = 200 µm). **b** Ferret diameter of 2K, 4K and 8K spheroids was estimated at day 1, 4 and 7 (n = 26; ***p < 0.005 relative to the 2K spheroid group). **c** Representative H&E histological images and scanning electron microscopic (SEM) images of 2K, 4K and 8K ASC spheroids (scale bar = 50 µm). **d** Second harmonic generation images of ASC sheet after 7 days of culturing (scale bar = 50 µm). **e** Representative SEM images of ASC sheets in top view and cross section (scale bar = 20 µm)

By adjusting the cell seeding density and the surface topography of the micropatterned agarose films, we were able to produce ASC spheroids of similar size in a high-throughput fashion. On day 1 of culture, ASCs with different seeding densities started forming spheroids of various sizes in the microwells. During in vitro culture, the measured spheroid diameter decreased gradually in each group of ASC spheroids with different seeding densities (Fig. 1a). On day 7, when the initial cell densities were 2 × 10^3^ (2K), 4 × 10^3^ (4K) and 8 × 10^3^ (8K) cells/microwell, the Ferret diameter of ASC spheroids was 188.6 ± 17.3, 233.4 ± 18.1 and 263.4 ± 8.9 μm, respectively. At each time point, the Ferret diameter of the 4K and 8K group was significantly larger than the 2K group (*p* < 0.005 respectively; Fig. 1b). A previous study showed that oxygen tension gradient varied less than 10% from the outer surface within the largest MSC spheroids up to 350 µm in diameter [23]. Therefore, we opted for a seeding density of 8K ASCs per microwell for spheroid generation without a concern of spheroid core necrosis.

Cell spheroids were subjected to histological examination of their cross sections. H&E staining showed abundant ECM deposition within the interstitial voids of the cell spheroids. Under electron microscope, cells on the top surface of the spheroids appeared to aggregate together tightly, and the cellular boundary was difficult to distinguish. The bottom part of the spheroids had adhered to the µ-slides with cells spreading out (Fig. 1c). As for ASC sheets, collagen deposition was noted in the interstitial spaces of cells under second harmonic imaging microscopy (Fig. 1d). Under electron microscopy, ASC sheets exhibited abundant matrix formation, and the cellular boundary appeared obscure on the sheet surface (Fig. 1e).

### Gene ontology functional analysis

**Fig. 2:**
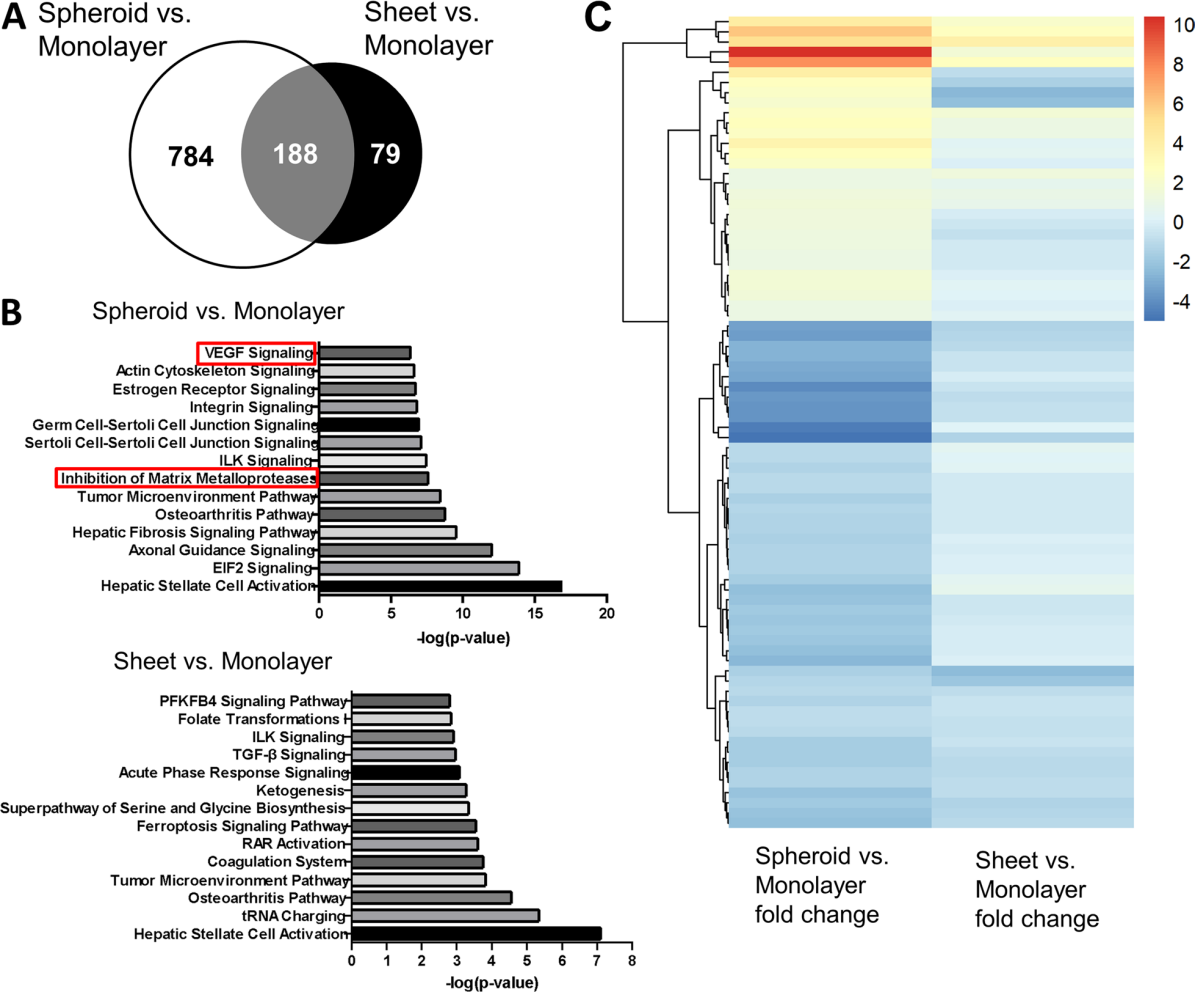
Analysis of RNA sequencing data in ASC spheroids and sheets relative to monolayer ASCs. **a** Venn diagram of the number of differential expression genes (> 2x) in spheroid and sheet groups compared to the monolayer group. **b** Functional analysis of pathways with significant differential expression genes in spheroid and sheet groups. Two angiogenesis-related pathways were found in the spheroid group (red square), but none was identified in the sheet group. **c** Angiogenesis-associated genes with differential expression between the spheroid and monolayer groups (> 2x) were selected to generate a heatmap using the log2 fold change values relative to the monolayer group

To better understand the biological characteristics affected by spheroid or sheet culture in ASCs, we analyzed the differential expression level of genes by RNA sequencing. Relative to monolayer ASCs, genes with more than twofold changes were identified in ASC spheroids and sheets. While 267 genes were found in the ASC sheet group, 972 genes were identified in the spheroid group, with 188 genes overlapped (Fig. 2a). Moreover, we used IPA for canonical pathway analysis to conduct functional enrichment analysis of the differentially expressed genes associated with the biological function of ASCs (Fig. 2b). The differentially expressed genes were enriched in regulation of the epithelial–mesenchymal transition by growth factors pathway (*p* = 1.53E−4), angiogenesis signaling pathway (*p* = 2.53E−4), regulation of the epithelial–mesenchymal transition pathway (*p* = 9.58E−4), HGF signaling (*p* = 2.76E−2) and FGF signaling (*p* = 2.92E−2). The raw sequencing data were submitted to the NCBI Sequence Read Archive with BioProject ID PRJNA742860. We further identified 80 angiogenesis-related genes with more than twofold differential expressions in the spheroid group relative to the monolayer group, and they were further analyzed to demonstrate the difference in their expression pattern between spheroid and sheet groups using a heatmap (Fig. 2c). These angiogenesis-related genes and their relative expression levels are shown in Additional file 1: Table S2.

### Angiogenesis-related gene expression in ASC spheroids and sheets

**Fig. 3:**
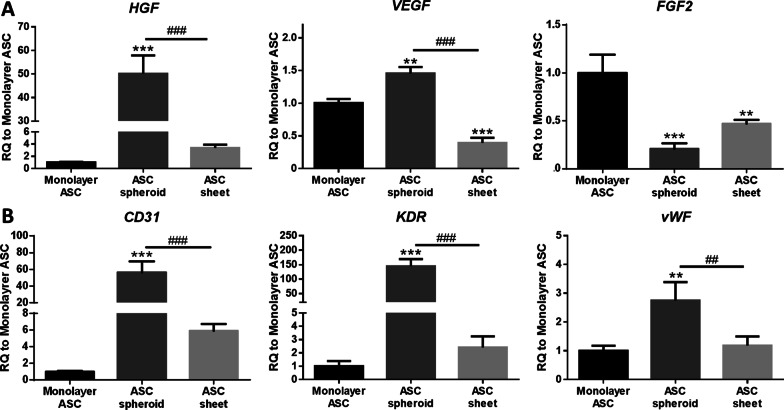
Angiogenesis-related gene expression of monolayer ASC, ASC spheroid and ASC sheet. **a** Angiogenic growth factors *HGF*, *VEGF*, and *FGF2*. **b** Mature endothelial markers *CD31*, *KDR* and *vWF*. The gene expression was normalized to monolayer ASC (n = 3; *p < 0.05, **p < 0.01, ***p < 0.005 from the monolayer group; ^##^*p* < 0.01, ^###^*p* < 0.005 between the indicated groups)

The relative mRNA expression of several angiogenic markers in ASC spheroids and sheets was analyzed by real-time qPCR. Relative to the ASC sheet group, the transcription numbers of angiogenic growth factors, including VEGF and HGF, were significantly higher in the ASC spheroid group (*VEGF*: 1.5 ± 0.1-fold vs. 0.4 ± 0.1-fold, *p* < 0.005; *HGF*: 50.1 ± 7.8-fold vs. 3.4 ± 0.5-fold, *p* < 0.005), while *FGF2* downregulation was noted in both ASC spheroid and sheet relative to the monolayer group (0.2 ± 0.1-fold and 0.5 ± 0.0-fold, respectively; Fig. 3a). The relative mRNA expression of mature endothelial cell markers, including platelet and endothelial cell adhesion molecule 1 (CD31/PECAM1), vascular endothelial growth factor receptor 2 (KDR/VEGFR2) and von Willebrand factor (vWF), was also evaluated. Relative to the monolayer group, *CD31*, *KDR* and *vWF* were all upregulated in ASC spheroids (*CD31*: 56.1 ± 13.5-fold, *p* < 0.005; *KDR*: 144.7 ± 24.5-fold, *p* < 0.005; *vWF*: 2.8 ± 0.6-fold, *p* < 0.01; Fig. 3b), but none was upregulated in ASC sheets.

### Fabrication of ASC spheroid sheets

**Fig. 4:**
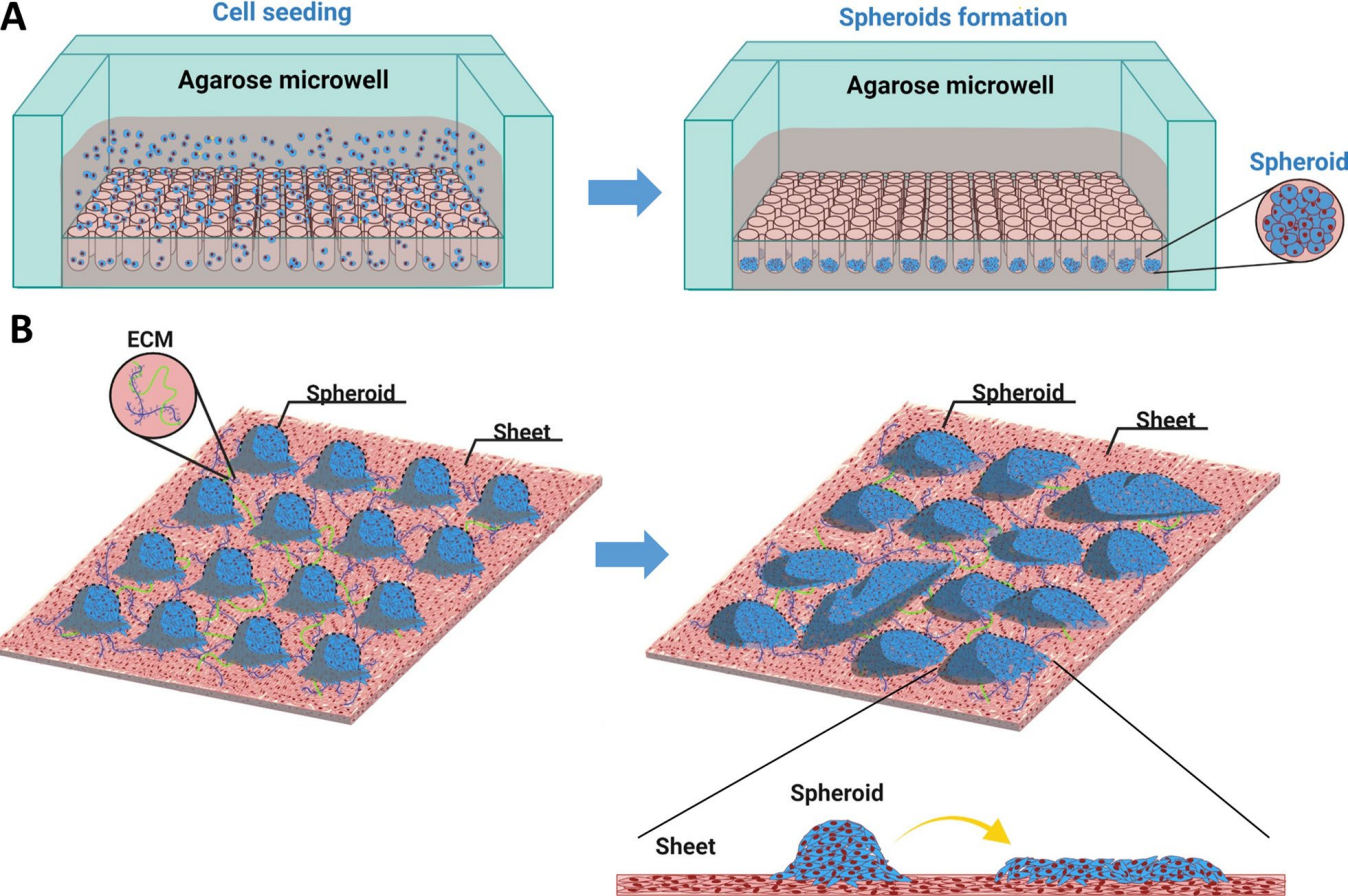
Schematic diagram of the manufacturing process of a composite ASC spheroid sheet. **a** Seeding ASCs in agarose microwells for spheroid formation. **b** Transferring ASC spheroids onto ASC sheets for spheroid sheet fabrication

**Fig. 5:**
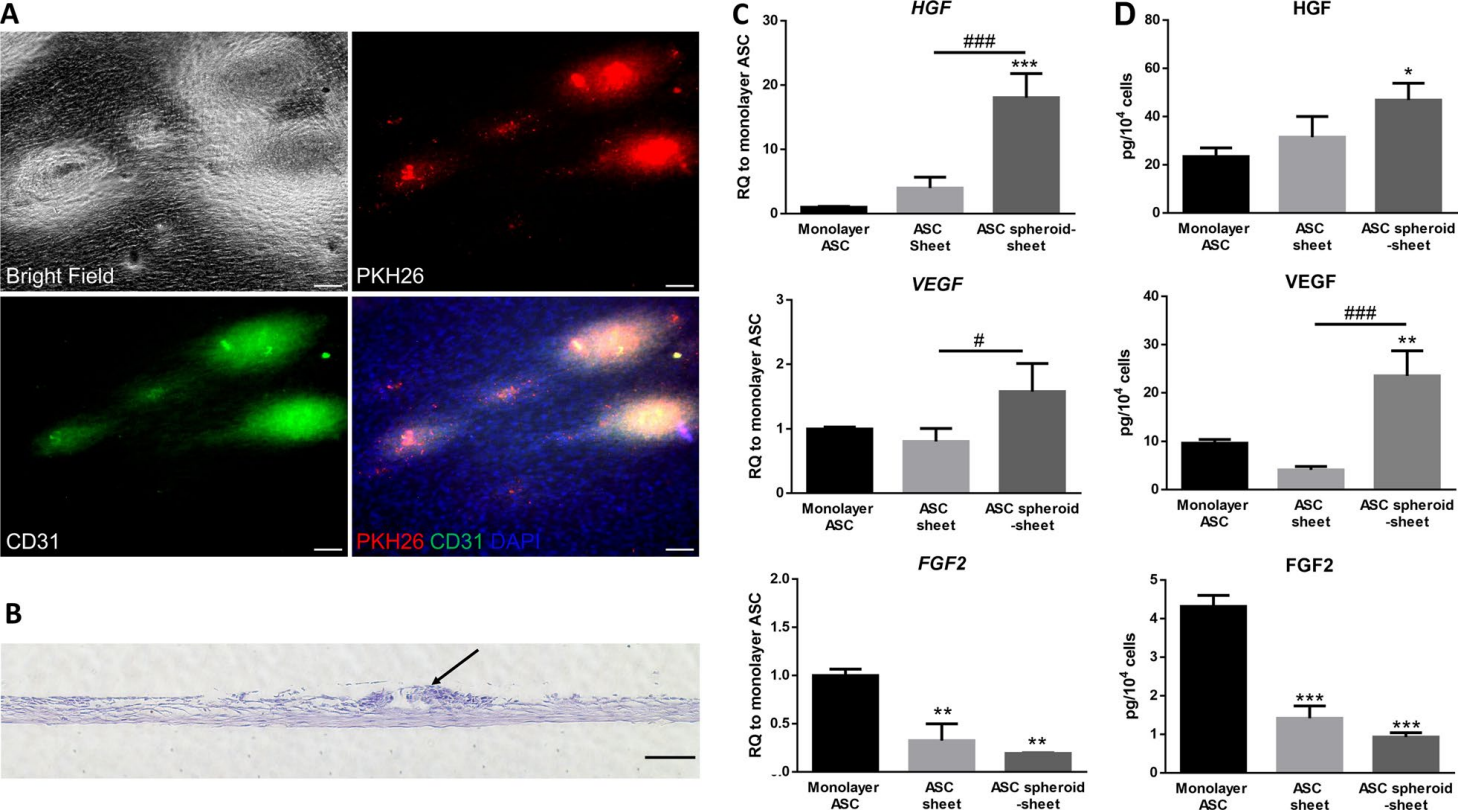
Expression of endothelial markers and angiogenic growth factors in ASC spheroid sheets. **a** CD31 immunofluorescence of the PKH26-labeled ASC spheroids seeded on ASC sheets. **b** Representative H&E histological image of the cross section of a ASC spheroid sheet (arrow indicates a seeded ASC spheroid; scale bar = 100 µm). **c** Gene expression analysis of *HGF*, *VEGF* and *FGF2* in monolayer ASC, ASC sheet and ASC spheroid sheet. The gene expression was normalized to monolayer ASC. **d** Release of HGF, VEGF and FGF2 from monolayer ASC, ASC sheet and ASC spheroid sheet into the medium as determined by ELISA. (n = 3; *p < 0.05, **p < 0.01, ***p < 0.005 from the monolayer ASC group; ^#^*p* < 0.05, ^###^*p* < 0.005 between the indicated groups)

A schematic diagram of the process of fabricating ASC spheroids and transferring spheroids for composite spheroid sheet formation is depicted (Fig. 4). PKH26-labeled ASC spheroids were employed to elucidate the process of transferring spheroids onto ASC sheets for composite spheroid sheet formation. The expression of mature endothelial marker CD31 was also evaluated. After **s**eeding the labeled ASC spheroids on ASC sheets, CD31 immunofluorescence showed co-localization of the PKH26 and CD31 signals on the surface of ASC sheets, suggesting the high contribution of ASC spheroids to CD31 expression (Fig. 5a). In the cross section of ASC spheroid sheets, H&E staining showed 2 or 3 layers of ASCs within the cell sheets with abundant ECM formation. The seeded spheroids over the sheet surface could be identified based on their high cellular density (Fig. 5b).

### Angiogenic growth factor expression in ASC spheroid sheets

The relative mRNA expression of angiogenic growth factors HGF, VEGF and FGF2 was analyzed by qPCR. After ASC spheroids were seeded on cell sheets, the ASC spheroid sheet group exhibited significant higher expression levels of *HGF* and *VEGF* relative to ASC sheets (*p* < 0.005 and *p* < 0.05, respectively; Fig. 5c). ELISA analysis of HGF levels in the conditioned medium revealed a significantly higher content of HGF released from ASC spheroid sheets relative to monolayer ASCs (46.8 ± 7.0 pg/10^4^ cells vs. 23.3 ± 3.7 pg/10^4^ cells, *p* < 0.05). ELISA analysis also revealed a significantly higher content of VEGF released from ASC spheroid sheets (23.5 ± 5.2 pg/10^4^ cells, *p* < 0.01 relative to monolayer ASC and *p* < 0.005 relative to ASC sheet; Fig. 5d).

### Tube formation assay of endothelial cells

**Fig. 6:**
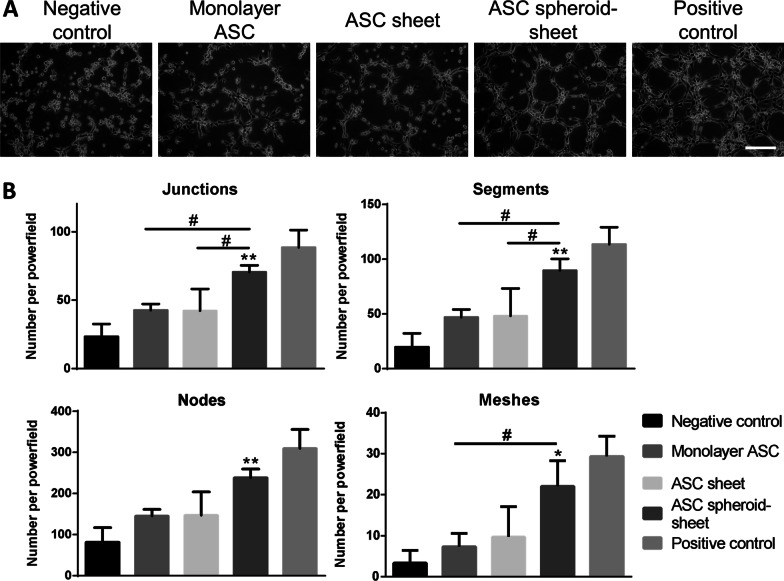
Effect of conditioned medium collected from monolayer ASCs, ASC sheets and spheroid on sheet cultures on the in vitro tube formation of HUVECs. **a** Representative microscopic images of endothelial cells after incubation with respective conditioned medium-supplemented EBM. HUVECs cultured in EGM2 served as a positive control, and those in DMEM-HG-supplemented EBM served as a negative control (n = 3). **b** Branching nodes, junctions, meshes and segments per power field were compared among different groups except the positive control. (*p < 0.05, **p < 0.01 from the negative control group; ^#^*p* < 0.05 between the indicated groups)

Conditioned media harvested from monolayer ASCs, ASC sheets and spheroid sheets were used for HUVEC culture, and the endothelial cells began to form a vascular network structure within 4 h (Fig. 6a). The in vitro tube formation of HUVECs was quantified by counting the total number of branching junctions, nodes, segments and meshes per power field. Relative to the negative control group, the spheroid sheet group exhibited significantly more tubelike structures in all parameters. Relative to the group receiving conditioned medium of ASC sheets, the spheroid sheet group also exhibited significantly more junctions (70.7 ± 4.9 vs. 42.3 ± 16.0 per power field, p < 0.05) and segments (89.7 ± 10.7 vs. 48.3 ± 25.0 per power field, p < 0.05; Fig. 6b).

### ASC spheroid sheets enhanced angiogenesis in CAM assay

**Fig. 7:**
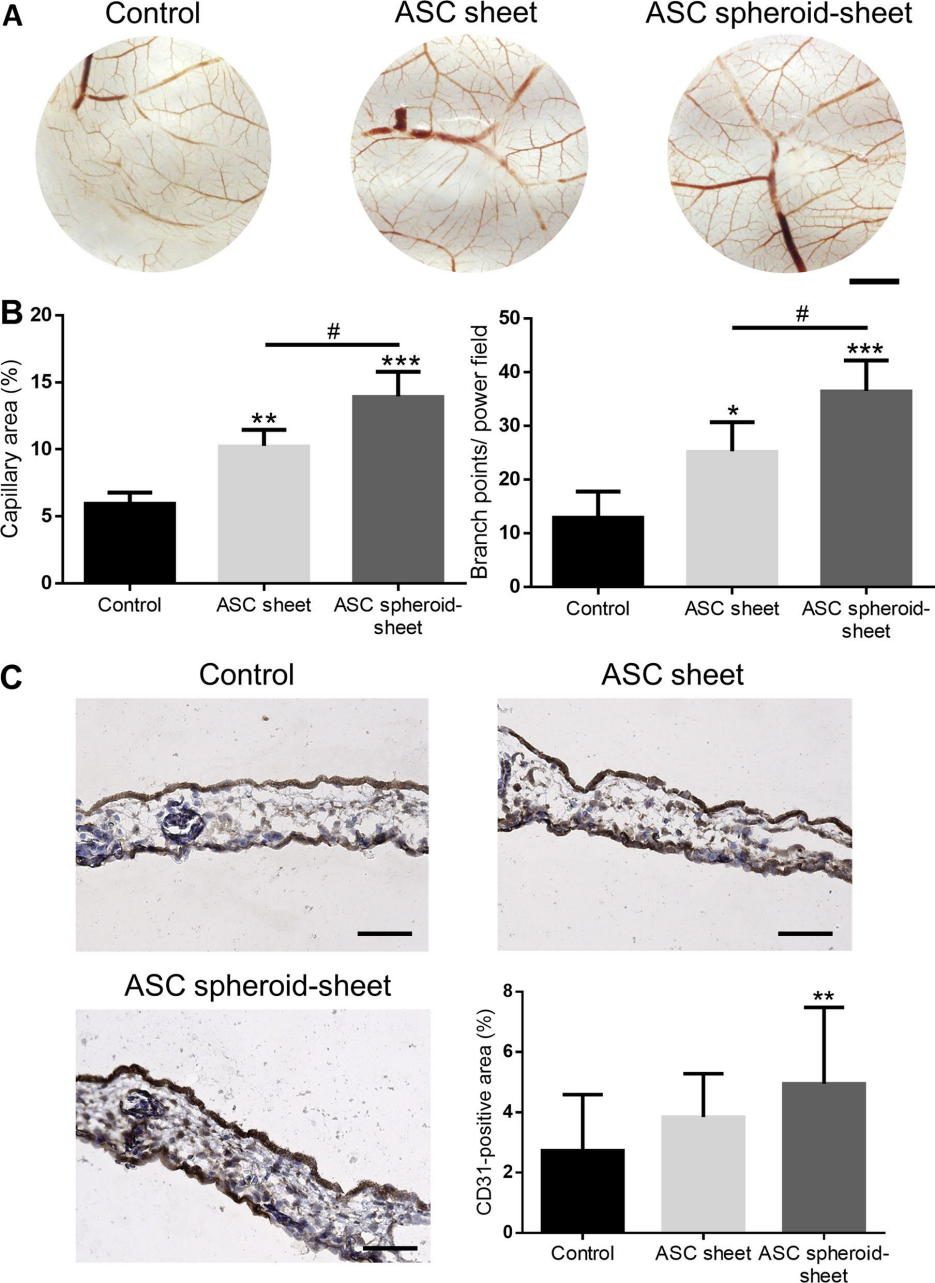
Chick embryo chorioallantoic membrane (CAM) assay. **a** Photographs of CAMs after treatment with ASC sheets or ASC spheroid sheets, which were loaded in the center of the power field. After 72 h of incubation, CAMs were excised and photographed (scale bar = 2 mm). **b** Blood vessel formation on CAMs was quantified by measuring the area covered by capillaries and counting the number of blood vessel branch points. **c** Immunohistochemical staining of endothelial marker CD31 in the CAM sections (scale bar = 50 μm). The ratio of CD31-positive area was significantly larger in the group of ASC spheroid sheet relative to the control. (n = 4; *p < 0.05, **p < 0.01, ***p < 0.005 compared to the control; ^#^*p* < 0.05 between the indicated groups)

We examined the capillary formation on CAMs to investigate the angiogenesis potential of ASC sheets and spheroid sheets (Fig. 7a). After implantation of ASC sheets or spheroid sheets on CAMs, we found significantly higher capillary area proportion around the cell constructs (ASC sheet: 10.3 ± 1.2%, spheroid sheet: 14.0 ± 1.8% vs. control: 6.0 ± 0.8%, *p* < 0.01 and *p* < 0.005, respectively) and significantly more blood vessel branch points (ASC sheet: 25.3 ± 5.4, spheroid sheet: 36.5 ± 5.7 vs. control: 13.0 ± 4.8 per power field, *p* < 0.05 and *p* < 0.005, respectively) relative to the control. The capillary area and branch points also exhibited significant difference between the groups of ASC sheet and ASC spheroid sheet (*p* < 0.05, respectively; Fig. 7b). Moreover, a significantly higher ratio of CD31-positive area in the CAM sections of the ASC spheroid sheet group was observed by immunohistochemistry (CD31-positive area: 5.0 ± 2.5% vs. 2.7 ± 1.9% of the control group, *p* < 0.01; Fig. 7c).

## Discussion

Tissue regeneration commonly requires blood flow to supply oxygen and nutrition and to remove waste. Capillary angiogenesis into ischemic tissue is therefore vital in relieving ischemia and promoting regeneration. ASC-based cell therapy represents a novel and promising strategy for therapeutic angiogenesis [24]. Although ASC sheets have been shown to display favorable effects in various tissue healing applications [8], downregulation of *VEGF* upon ASC sheet formation can be detrimental to their application for therapeutic angiogenesis [14]. In this study, successful fabrication of spheroid-augmented ASC sheets was achieved, which demonstrated superior angiogenic potential compared to ASC sheets alone. Taking advantage of the enhanced gene expression of angiogenic growth factors and endothelial markers after ASC spheroid formation, the spheroid sheet composite system demonstrated enhanced expression of these angiogenesis-related factors relative to ASC sheet alone. Co-delivery with recombinant growth factors or gene transfection had been applied to circumvent this disadvantage of ASC sheet therapy previously [13, 14]. In comparison with these maneuvers, our approach of combining ASC spheroids and sheets employed no exogenous biological or genetic material, thus significantly decreasing the regulatory hurdles for clinical translation.

Technology of 3D culture has been applied as tissue-engineered products to exploit their tissue regenerative effects in recent years. Comparing to monolayer culture, culturing ASCs as 3D aggregates causes substantial changes in the pattern of gene expression [15–17]. Many reports have shown that aggregation of ASCs as spheroids or sheets can enhance their therapeutic potentials [15, 16, 25]. The additional dimensionality of 3D cultures leads to different cellular responses because of its influences on the physical interactions with the surrounding cells and the spatial organization of the cell surface receptors. The changes of these spatial and physical aspects resulting from 3D cultures affect the signal transduction of cells, resulting in altered gene expression and cellular behavior [5, 20]. Previously, we found enhanced expression of stemness markers, including Oct4, Sox2 and Nanog, in ASCs within cell spheroids and sheets [5, 9], but the gene expression level of angiogenesis growth factors was differentially regulated upon ASC spheroid or sheet formation [10, 15]. In this study, transcriptomic analysis of ASC spheroids and sheets using RNA sequencing showed substantial different profiles of gene expression, and we confirmed that angiogenesis-related genes are predominantly upregulated in ASC spheroids, but not in ASC sheets.

Although the underlying mechanism of enhanced regenerative capabilities in different 3D-cultured ASCs has not been thoroughly elucidated, there is convincing evidence showing that the cellular microenvironment plays an essential role in determining the stem cell properties [11, 26]. One obvious change upon aggregation of ASCs is the development of a hypoxic environment in the core of each spheroid. However, cellular metabolism in spheroids significantly decreased as the number of cells and resultant spheroid size increased, thus preventing cellular death in the hypoxic core of cell spheroids [23]. In addition to the hypoxic milieu, the enhanced function of ASC spheroids is associated with the morphology change of cells [27]. For example, the relaxation of cytoskeleton tension of MSCs in 3D spheroids was critically associated with the expressional upregulation of an important pluripotency marker Nanog [28]. On the contrary, the favorable effects of ASC sheets on cells are attributed to the stimulatory effect of A2-P on collagen synthesis during in vitro culture. ECM within cell sheets has been shown to play an important role in modulating the biological properties of stem cells [10, 11]. The critical role of the ECM niche and its component proteins has been highlighted in determining the endothelial lineage commitment of resident MSCs [29]. Hence, the different capabilities of secreting angiogenic growth factors and endothelial differentiation may be attributed to the different composition of ECM proteins within ASC spheroids and sheets.

The conventional techniques for cell spheroid formation involve the use of physical stimuli to promote cell aggregation, such as centrifugal force, electric and magnetic field, and ultrasound [26]. One of the most commonly used techniques is the hanging drop method, which allows gravity to facilitate cellular aggregation. This method enables the reliable generation of uniform cell spheroids, but it is relatively labor-intensive and prone to pipetting error [26]. Recently, coating multi-well plates with non-adherent polymer hydrogels has evolved to be an efficient approach to impair cell adhesion to surfaces and stimulate cell aggregation to generate homogenous cell spheroids [20]. We adopted this method in the current study because it provides a straightforward procedure to obtain plentiful standardized cell spheroids, and the size of the cell spheroids can be adjusted by controlling the cell seeding density. As for ASC sheet fabrication, temperature-responsive culture plates have been used to obtain cultured cells and their deposited ECM as intact sheets [8]. However, the entire harvesting process is relatively complicated, time-consuming, and requires special culture substrates. Alternative approaches of using derivatives of ascorbic acid, such as A2-P, enable creating ASC sheets without the use of thermosensitive culture plates [9]. Ascorbic acid plays a key role in the biosynthesis of collagen and other ECM constituents [30], so supplementing A2-P in the culture medium can generate reasonably thick ASC sheets that allows easy transplantation to the injury sites [10].

Cell spheroids and cell sheets exhibit unique biological characteristics and advantages in transplantation, respectively. Transition of these two common forms of scaffold-free 3D culture had been attempted to maximize the advantages of 3D culture constructs. For example, a method for harvesting uniformly sized multicellular human dermal fibroblast spheroids was developed by self-assembly of microscaled cell sheets [31]. Likewise, size-controllable MSC spheroids had been fabricated from microscaled cell sheets for regenerative applications [32]. Conversely, ASC spheroids were seeded on a temperature-responsive hydrogel to fabricate cell sheets in another study [33]. In comparison with the transition between cell spheroids and cell sheets, the combination of the two forms of 3D culture can yield substantial advantages, including shortened culture time, more robust 3D matrix formation and easy cell transplantation. Notably, a previous study employed mouse myoblasts to prepare a composite sheet containing human MSC spheroids. Combining MSC spheroids increased the survival rate and decreased the inflammatory response associated with allogeneic immune rejection toward myoblast sheets [34]. Thus, 3D-cultured ASC spheroid sheet composite technology may also provide immune evasion potential and improve the outcome of ASC allo-transplantation.

It may be argued that ASC spheroids alone can deliver sufficient pro-angiogenic effects without co-transplantation with ASC sheets. However, the delivery of ASC spheroids to the injury sites may be difficult, so certain biomaterials have been developed to facilitate the transplantation of ASC spheroids [16, 20]. For example, ASC spheroids in hydrogels exhibited good proliferative activity and differentiation potential [20], and a thin poly-D,L-lactic acid film was developed as a carrier of ASC spheroids to enhance engraftment [35]. Although such approaches exhibit certain advantages, the addition of exogenous biomaterials may also cause untoward responses upon cell transplantation. The current approach using cell sheets of the same source as the carrier of ASC spheroids for therapeutic purposes allows a scaffold-free maneuver throughout the 3D culture and cell delivery process, thus minimizing the interference of exogenous biomaterials. Further animal studies are required to fully validate the therapeutic effects resulting from the enhanced angiogenic potential in ASC spheroid sheets.

## Conclusion

In this study, we fabricated size-controlled ASC spheroids using micropatterned agarose hydrogels, and the spheroids were further seeded onto ASC sheets to engineer spheroid sheet composites. The transcriptomic profile of ASC spheroids and sheets by RNA sequencing revealed significant differences in the gene expression pattern, with upregulation of angiogenesis-related genes noted only in ASC spheroids. When the ASC spheroids adhered to the sheet surface, outward migration of the cells and strong CD31 expression was observed in these cells. Moreover, enhanced expression of angiogenic growth factors was noted in ASC spheroid sheets relative to ASC sheets. Consequently, conditioned medium of ASC spheroid sheets significantly enhanced tube formation of endothelial cells in vitro. CAM assay in chick embryo also showed significantly higher capillary area, more branch points and more CD31-positive area after applying spheroid sheets. Hence, overlaying cell spheroids can enhance the angiogenic capabilities of ASC sheets without chemical or genetic manipulation. Meanwhile, cell sheets in this composite cell construct also act as ideal carriers to facilitate the delivery of ASC spheroids to the tissue injury site. These two formats of scaffold-free 3D-cultured ASCs complement each other to yield a novel combination of engineered cell constructs, which exhibit great potential to enhance ischemic tissue regeneration.

## Supplementary Information

**Additional file 1:** **Table S1.** Primer sequences used for the real-time qPCR analysis. **Table S2.** The angiogenesis-related genes and their relative expression levels in the ASC spheroid and sheet groups.

## Data Availability

Most data generated or analyzed in this study are included in this article and a publicly accessible repository. The other data that support the findings of this study are available from the corresponding author upon reasonable request.

## Abbreviations

ASC: Adipose-derived stem cells
A2-P: Ascorbic acid 2-phosphate
3D: Three-dimensional
MSC: Mesenchymal stem cells
ECM: Extracellular matrix
ELISA: Enzyme-linked immunosorbent assay
HGF: Hepatocyte growth factor
VEGF: Vascular endothelial growth factor
FGF: Fibroblast growth factor
vWF: Von Willebrand factor
HUVEC: Human umbilical vein endothelial cell
EBM: Endothelial basal medium
EGM: Endothelial growth medium
CAM: Chorioallantoic membrane
