# Supplementary material for: Enhanced angiogenic potential of adipose-derived stem cell sheets by integration with cell spheroids of the same source
Source: Stem Cell Res Ther. 2022 Jun 28;13:276. doi: 10.1186/s13287-022-02948-3 (PMC9241243; doi:10.1186/s13287-022-02948-3)
Supplement: Supplementary file 1 — Additional file 1: Table S1. Primer sequences used for the real-time qPCR analysis. Table S2. The angiogenesis-related genes and their relative expression levels in the ASC spheroid and sheet groups. [file 13287_2022_2948_MOESM1_ESM.docx]

**Supplementary Material**

**Table 1.** Primer sequences used for the real-time qPCR analysis

| Target gene | Primer sequence | |
| --- | --- | --- |
| *HGF* | Forward | 5’-CTAGATCTTTCCAGTTAATCACACAAC-3’ |
|  | Reverse | 5’-TTCGGAGTCAGTGCCTAAAAGAG-3’ |
| *VEFG* | Forward | 5’-CTTGCCTGGCTGCTCTAC-3’ |
|  | Reverse | 5’-CACACAGGATGGCTTGAAG-3’ |
| *FGF2* | Forward | 5’-CCAAGCGGCTGTACTGCAAA-3’ |
|  | Reverse | 5’-TCCTTGACCGGTAAGTATTG-3’ |
| *CD31* | Forward | 5’-TCTATGACCTCGCCCTCCACAAA-3’ |
|  | Reverse | 5’-GAACGGTGTCTTCAGGTGGTATTTCA -3’ |
| *KDR* | Forward | 5’-ACTTTGGAAGACAGAACCAAATTATCTC-3’ |
|  | Reverse | 5’-TGGGCACCATTCCACCA-3’ |
| *vWF* | Forward | 5’-TGCTGACACCAGAAAAGTGC-3’ |
|  | Reverse | 5’-AGTCCCCAATGGACTCACAG-3’ |

**Table 2.** The angiogenesis-related genes and their relative expression levels in the ASC spheroid and sheet groups

| **Gene name** | **Spheroid vs. Monolayer  fold change** | **Sheet vs. Monolayer  fold change** |
| --- | --- | --- |
| *MMP9* | 1341.880026 | 3.341465644 |
| *ACTG1* | -2.193436909 | 1.077317568 |
| *HGF* | 214.7713907 | 5.934374538 |
| *PLXDC1* | 63.04835449 | 5.938109468 |
| *ANXA2* | -2.670301857 | 1.033999312 |
| *ARHGAP22* | 2.100232315 | 1.019470271 |
| *SFRP2* | 32.26357604 | 14.08835357 |
| *SERPINF1* | 17.63230018 | 4.277347624 |
| *EDNRA* | 15.86038952 | -1.955656128 |
| *AGT* | 11.34428841 | 1.240580162 |
| *COL8A2* | 2.10378535 | 2.705650511 |
| *CXCL12* | 2.640460971 | -1.135769955 |
| *MDK* | 7.008275846 | 2.083679331 |
| *COL18A1* | 6.804919554 | 1.298250022 |
| *ANGPTL4* | 6.292860748 | -2.875236889 |
| *EMILIN1* | 2.474619916 | 1.867954314 |
| *ENPP2* | 2.526170402 | -1.35415557 |
| *EPAS1* | -2.551315264 | -1.588694744 |
| *PLK2* | 5.397316627 | 2.133820572 |
| *DCN* | 5.197426594 | 3.175547149 |
| *EMP2* | 5.173698273 | 1.000648669 |
| *PGF* | 5.029746644 | -6.473091754 |
| *GADD45A* | -2.048418896 | -2.018601052 |
| *S1PR1* | 3.843914188 | -4.777941685 |
| *GLUL* | 2.087670448 | -1.288777133 |
| *HIF1A* | -2.496679907 | -1.188411012 |
| *PDGFRA* | 3.073849894 | 1.169844817 |
| *NFATC4* | 3.068049223 | 1.017454826 |
| *LGALS3* | 2.9964262 | 1.138453504 |
| *JUN* | 2.213704362 | -1.644840413 |
| *SRPX2* | 2.817050258 | 1.74387374 |
| *RRAS* | -2.656251707 | -1.224916711 |
| *ANPEP* | -2.688601525 | -2.160674085 |
| *RHOB* | -2.711130204 | -1.955829657 |
| *STAT1* | -2.724281458 | -1.221776859 |
| *MYH9* | -2.748261067 | 1.025284802 |
| *ID1* | -2.895267596 | -5.684469341 |
| *HSPB1* | -2.977803183 | -1.215466706 |
| *MMP2* | 2.100545832 | 1.193805043 |
| *CALD1* | -3.062253371 | 1.000514203 |
| *MYDGF* | -2.511524757 | -1.076501779 |
| *CLIC4* | -3.164430423 | -1.62291908 |
| *RTN4* | -3.19769433 | -2.209869418 |
| *NINJ1* | 2.288789751 | -1.269145185 |
| *CAV1* | -3.293232826 | -1.860491857 |
| *THY1* | -3.37483692 | 1.401944061 |
| *ITGA5* | -3.532188183 | -1.390481196 |
| *FN1* | -3.762900648 | -1.18278901 |
| *ATP2B4* | -3.794370197 | -2.50558917 |
| *FLNA* | -3.842422429 | -1.13092744 |
| *TNFRSF12A* | -3.991681783 | -1.249657149 |
| *PLXND1* | -2.020696118 | -1.665457286 |
| *PNPLA6* | -2.073017375 | -1.76165574 |
| *CREB3L1* | -4.451736218 | -1.371929304 |
| *ENG* | -4.583062493 | -2.490814951 |
| *VEGFC* | -4.690375669 | -1.822137173 |
| *PXN* | -2.017351117 | -1.500803281 |
| *ADM* | -4.799821514 | -2.243533626 |
| *GREM1* | -4.839950897 | -1.084507813 |
| *SPARC* | -5.265123331 | 1.53707979 |
| *ROCK2* | -2.037626485 | -1.238838487 |
| *RECK* | -6.110879757 | 1.030560405 |
| *MFGE8* | -6.901804712 | -2.048900838 |
| *HSPG2* | -7.060659859 | -1.583051517 |
| *COL4A2* | -8.383887841 | -1.630758868 |
| *FBLN5* | -9.472060926 | -1.098803378 |
| *SPRED1* | 2.161268116 | -1.314921484 |
| *ADAMTS1* | -10.19012779 | -2.570687652 |
| *SULF1* | -2.599775191 | 1.253558925 |
| *HSPB6* | -11.76364681 | -2.382571675 |
| *LOXL2* | -12.57395328 | -2.013034962 |
| *TGFBR2* | -2.214505484 | -1.200180266 |
| *THBS2* | -2.079222624 | 1.400337906 |
| *DDAH1* | -14.22798142 | -1.696999561 |
| *F3* | -14.57809962 | -1.765949007 |
| *COL4A1* | -17.3853714 | -1.529642916 |
| *THBS1* | -26.09199525 | 1.101493982 |
| *SERPINE1* | -32.50907013 | -2.730017445 |
| *VEGFB* | 2.039647761 | 1.560705873 |
| *WARS* | -2.350620175 | -4.239109093 |
